# Emerging Roles of Long Non-Coding RNAs in Renal Fibrosis

**DOI:** 10.3390/life10080131

**Published:** 2020-08-01

**Authors:** Jinwen Lin, Zhengqian Jiang, Chenxi Liu, Dawei Zhou, Jiayu Song, Yuxuan Liao, Jianghua Chen

**Affiliations:** 1 ZJU-UoE Institute, International Campus, Zhejiang University, Haining 314400, China; 11971004@zju.edu.cn; 2 Kidney Disease Center, the First Affiliated Hospital, College of Medicine, Zhejiang University, Hangzhou 310003, China; 3 ZJU-UoE Institute, The University of Edinburgh, Edinburgh EH8 9JU, UK; 4 Xiangya School of Medicine, Central South University, Changsha 410013, China; django@csu.edu.cn (Z.J.); lcx0929@csu.edu.cn (C.L.); zhoudw@csu.edu.cn (D.Z.); 8303180711@csu.edu.cn (J.S.); liaoyuxuan@csu.edu.cn (Y.L.)

**Keywords:** long non-coding RNA, fibrosis, gene therapy, myofibroblast, phenotype transition

## Abstract

Renal fibrosis is an unavoidable consequence that occurs in nearly all of the nephropathies. It is characterized by a superabundant deposition and accumulation of extracellular matrix (ECM). All compartments in the kidney can be affected, including interstitium, glomeruli, vasculature, and other connective tissue, during the pathogenesis of renal fibrosis. The development of this process eventually causes destruction of renal parenchyma and end-stage renal failure, which is a devastating disease that requires renal replacement therapies. Recently, long non-coding RNAs (lncRNAs) have been emerging as key regulators governing gene expression and affecting various biological processes. These versatile roles include transcriptional regulation, organization of nuclear domains, and the regulation of RNA molecules or proteins. Current evidence proposes the involvement of lncRNAs in the pathologic process of kidney fibrosis. In this review, the biological relevance of lncRNAs in renal fibrosis will be clarified as important novel regulators and potential therapeutic targets. The biology, and subsequently the current understanding, of lncRNAs in renal fibrosis are demonstrated—highlighting the involvement of lncRNAs in kidney cell function, phenotype transition, and vascular damage and rarefaction. Finally, we discuss challenges and future prospects of lncRNAs in diagnostic markers and potential therapeutic targets, hoping to further inspire the management of renal fibrosis.

## 1. Introduction

Renal fibrosis is a process of excessive deposition and accumulation of extracellular matrix (ECM) in renal parenchyma, due to the increased activity of myofibroblasts, which exhibits a typical final result of virtually all chronic and progressive kidney diseases. In the process of chronic injury, the fibrous matrix is over deposited, which destroys the original structure of the kidney and compresses arteries at the same time, resulting in insufficient blood supply and ultimately leading to renal failure [1]. Whatever the prime causes, renal fibrosis represents a pathological injury to the kidney following a routine wound healing process after prolonged and sustained damage. During renal fibrosis, the deposition of the fibrous matrix is essentially a gradual process, which is represented by inflammation, activation, and migration of myofibroblast, and consequently ECM deposition and remodeling. Several cellular processes, such as intrinsic renal fibroblast-type cells activation and epithelial-mesenchymal transition (EMT), have been elucidated as significant mechanisms to produce fibroblast phenotypes that cause fibrous deposition under pathological conditions. Complex fibrogenic factors are involved in the pathogenesis of renal fibrosis. Therein, transforming growth factor-β (TGF-β) is regarded as a critical mediator in renal fibrosis through the activation of both Smad-based and non-Smad-based signaling pathways. Recent discoveries on an intricate set of coactions between TGF-β/Smad signaling and others, known as pathway crosstalk, however, add a new level of complication in the molecular regulation of renal fibrosis [2]. Therefore, researchers turned their attention to the molecular mechanism, which is a potential solution to renal fibrosis by regulating the cell pathways.

As a representative of non-coding RNAs who make up the majority of the genome transcription production, lncRNAs were shown pervasively transcribed, while protein-coding genes account for only 2% [3]. Widely existed in eukaryotes, the functionality of this RNA molecule is constantly being discovered and remained controversial [4]. Researches have shown that lncRNAs serve as potential key regulatory factors in gene expression networks, including the significant proportion of the non-coding transcriptome and revealing a surprising range of sizes and shapes, which are receiving increasing interest. It has been demonstrated that lncRNAs contain versatile functions, including transcriptional regulation, nuclear domains organization, and regulation of proteins or RNA levels [5]. According to the role of lncRNAs at different levels in gene expression, it is remarkable that lncRNAs can act as regulator guides, enhancers, decoys, or scaffolds, especially during post-transcriptional and post-translational processes [6]. LncRNAs can achieve their biological effects in different layers, whose abnormal expression has been confirmed to be associated with a variety of pathological environments [7]. A growing amount of experimental studies inform that lncRNAs highly participate in the formation of renal fibrosis, and they are associated with different cell masses in each process of kidney disease [8]. Hence, the new correlation between lncRNAs and renal fibrosis has become a potential solution for organ fibrosis. However, which levels of gene expression are regulated by lncRNAs and the relevant molecular mechanisms are becoming complex, requiring a systematic review.

In recent years, the multiple roles of lncRNAs in renal fibrosis have made significant progress. In this review, the paper will start with a brief introduction to the biology of lncRNAs. Then current and emerging roles of lncRNAs as factors for renal parenchymal functions, phenotype transition, and vascular damage and rarefaction are summarized, along with their potential diagnostic power and recent advances in fibrosis treatments. Finally, the challenges and future prospects of lncRNAs are also assembled in order to look towards the future development of renal fibrosis treatments.

## 2. LncRNAs Biology

**Figure 1:**
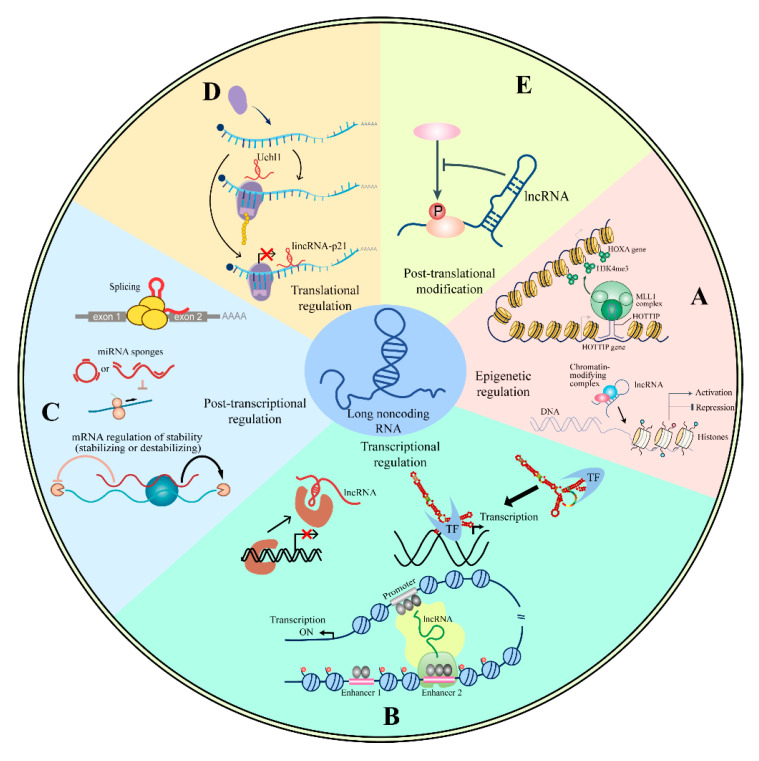
The schematic diagram illustrates the biological function of lncRNA in the regulation of gene expression. (**A**) LncRNAs unleash the potential of epigenetic gene regulation by recruiting the function of a generic suite of chromatin-modifying proteins to particular genomic loci either near or far away from the host gene. Recruitment of histone-modifying enzyme change chromatin states and further contribute to chromatin remodeling, whereas the methylation state of DNA can be altered by DNA methylases and demethylases. Regulating the condition of histones or DNA changes the approachability of the DNA template to the DNA-binding protein, thus facilitating or repressing selective gene expression. (**B**) LncRNAs are able to modulate gene expression at the transcriptional level by directly trapping a transcription factor, or any other proteins or complexes, which then participate in the formation of preinitiation complex and/or RNA polymerase II to either promote or suppress transcription. Moreover, enhance lncRNAs modulates transcription by interacting with the large transcriptional co-activating complex to bring the enhancer and the target gene into close proximity. (**C**) LncRNAs can alter gene expression at post-translational level through the regulation of alternative splicing, function as miRNA sponges to block the function of miRNA, or the recruitment of protein related to mRNA degradation to modulate mRNA stability. (**D**) Some lncRNAs are able to promote or inhibit translation by binding to the translational machinery. (**E**) LncRNAs are capable of regulating post-translational modification of proteins by disturbing the interaction between proteins and protein-modifying enzymes.

Eukaryotic RNA transcription and processing produce a variety of RNA molecules, among which lncRNAs have emerged as a major proportion of the eukaryotic transcriptome. LncRNAs can be broadly placed into six classes, including sense lncRNAs, antisense lncRNAs, intronic lncRNAs, bidirectional lncRNAs, long intergenic non-coding RNAs (lincRNA), and enhancer RNAs, on the bias of the genomic location from where the RNA transcript originates, as previously stated. More recently, the seemingly infinite discovery of novel long non-coding transcriptomes has remarkably widened the diversity of lncRNAs, and emerging lines of evidence identified a series of new lncRNA species with unexpected structures that are generated via unusual RNA processing pathways, such as circular RNAs, intron-derived long non-coding RNAs with snoRNA ends (sno-lncRNAs) [9,10,11], pre-mi-lncRNAs [12,13], etc. Defining lncRNAs merely on the basis of their genome position accompanied by processing pathways [14], however, is intellectually far from satisfying to illustrating its in-depth mechanism. Indeed, a meaningful comprehension of lncRNA function can merely be obtained from a detailed study in a case-by-case manner. Researchers have found that lncRNAs carry out their functions by directly interacting with DNA, RNA, and proteins, thereby affecting the regulation of the molecular level of cells. These above interactions can, thus, classify lncRNAs into enhancers, guides, decoys, and scaffolds [15]. Additionally, transcripts of lncRNAs are now known to participate in the epigenetic, transcriptional, post-transcriptional, translational, and post-translational regulation of gene expression in diverse cellular contexts and biological processes [16,17]. Although only a few functional lncRNAs have been established and rigorously evaluated to date, mechanistic principles were derived according to the current understanding of lncRNAs, as illustrated in Figure 1.

### 2.1. Epigenetic Regulation

In the nucleus, lncRNAs unleash the potential of epigenetic gene regulation by recruiting the function of a generic suite of chromatin-modifying proteins to particular genomic loci, thus, contributing to the modulation of chromatin states and further the chromatin remodeling, as well as the alterations of gene expression. The lncRNA-containing complexes can facilitate either selective gene activation or repression of specific genes, following the nature of the chromatin complex. For instance, several lncRNAs are observed to interact with histone H3K4 methyltransferases to activate gene expression, whereas others interact with the polycomb repressive complex 2 (PRC2), which leading to H3K27me3 and gene silencing [18]. Additionally, individual lncRNAs have been demonstrated to recruit DNA methyltransferases (DNMTs), such as DNMT1 and DNMT3b, to catalyze the generation of the DNA methylation, thereby leading to the establishment of genomic methylation patterns [19,20].

Several emergent themes of lncRNA-directed chromatin modification were first obvious within the genomic imprinting phenomena, by which gene expressed in a mutually exclusive, parent-of-origin, allele-specific manner. Indeed, detailed analysis has demonstrated that lncRNA-directed imprinting must flatly be performed in cis, due to the disparity of epigenetic conditions of the two alleles existing in the same nuclear environment. A cardinal example of imprinted lncRNA-mRNA is the paternal-specific gene clusters lncRNA Air; an antisense transcript originated from the insulin-like growth factor 2 receptor gene in a bunch of imprinted, maternally expressed protein-coding genes in mammals. The promoter of Air located within a CpG island that is not methylated on the paternal allele, resulting in the parental transcription of Air, which then targets epigenetic modifiers G9a to silences the imprinted locus [21]. In contrast, the maternal allele is hypermethylated, such that Air is silenced on the maternal chromosome, thus, enabling the transcription of flanking protein-coding genes [22]. A similar study also reported on the role of lncRNA Kcnq1ot1 at the Kcnq1 locus for autosomal imprinting [23]. Apart from autosomal imprinting, lncRNA Xist functions in cis to facilitate X inactivation by recruiting and interacting with diverse proteins, a mechanism that virtually refers to chromosome-wide epigenetic imprinting, such that activate histone deacetylase 3, the deacetylation of histones or exclude RNA polymerase II [24]. An intriguing fact is that mechanisms for lncRNA-directed chromatin modification not only participate in epigenetic silencing in cis (but also in trans), where for instance, the HOTAIR lncRNA provides surfaces to assemble particular histone-modifying enzymes, thereby targeting gene silencing and histone marks to specific genes in the Hox cluster [18].

### 2.2. Transcriptional Regulation

Accumulating evidence identified the active role of lncRNAs on transcription, suggesting an additional mode of regulation in gene expression. Indeed, the chances are that a lncRNA can trap and regulate a transcription factor, or any other proteins or complexes involved in gene expression, which then participate in the formation of preinitiation complex and/or RNA polymerase II to either promote or suppress transcription. GAS5 was observed to interact with the DNA-binding domain of the activated transcription factor glucocorticoid receptor to compete with its interaction with glucocorticoid response elements, such that decrease the expression of the glucocorticoid-responsive genes [25]. In contrast, lncRNAs can also capture specific RNA-binding transcription factors at gene regulatory elements to sustain transcription occupancy, thereby contributing to the stability of the gene expression programs [26]. Besides, some lncRNAs function as decoys for molecules that negatively regulate gene expression. A Box H/ACA snoRNA-ended lncRNA SLERT, for example, directly interacts with the DEAD-box RNA helicase DDX21 via a 143-nt non-snoRNA sequence, thereby counteracting the suppressive effect of DDX21 molecules on RNA Pol I and terminally promoting pre-rRNA transcription [10].

Besides, a group of lncRNAs that share similarity with activating ncRNAs, regulate DNA looping and chromatin enhancement through the formation of a large transcriptional co-activating complex termed Mediator. A prime example is enhancer RNA (eRNA), which contacts Mediator subunits to facilitate the formation of chromosomal looping thereby bringing the enhancer and the target gene into close proximity, as corroborated by chromosome conformation capture [27]. Moreover, it has been proved that in some cases, the act of lncRNA transcription alone instead of the lncRNA product is sufficient to confer a regulation function [28]. By way of illustration, Kelly M Anderson shows blockade of lncRNA upperhand transcription, rather than knockdown of the mature transcript, prevented the super-enhancer signature and elongation of RNA polymerase II through the Hand2 enhancer locus, thereby abolished neighboring protein-coding gene Hand2 expression [29].

### 2.3. Post-Transcriptional Regulation

*Splicing:* Nuclear speckles are dynamic structures located in the interchromatin domains, consisting of pre-mRNA splicing factors, such as spliceosome subunits, serine-/arginine-rich proteins, as well as small nuclear ribonucleoproteins. In fact, emerging data promotes that speckles are sites from where splicing factors are recruited to the locus of active transcription [30]. MALAT1 simultaneously interacts with actively transcribed gene bodies, and multiple splicing-associated proteins consist of nuclear speckles [31]. Moreover, silencing of MALAT1 change the localization together with the activity of splicing factors, such that alters the pattern of alternative splicing for a set of nascent transcripts [32], thereby hinting at a central role for MALAT1 as a scaffold that direct the positioning of splicing machinery at the active sites of gene transcription

*Sponges for miRNA:* miRNA exerts functions by annealing to complementary sites on the coding or specific sequences in the 3′ UTR of the targeted gene transcripts, such that facilitates recruitment of protein complex to either repress translation or promote degradation of the targeted mRNA [33]. Several lines of evidence brought forth to the idea that a category lncRNA that characterized by tandem repeats of miRNA-binding sites, have the biological function as miRNA sponge through sequestering miRNA from their endogenous targets, thereby abolishing their suppression on gene expression [34]. For example, the linc-MD1 contacts and sequesters the miR-133, and as a result, blunts miR-399 action and changes the expression of MAML1 and MEF2C, thereby controlling the differentiation of human myoblasts [35].

*mRNA stability:* mRNA stability can be manipulated in order to control its half-life. Apart from function as miRNA sponges to indirectly regulate mRNA stability, emerging evidence unleash an additional player of lncRNA in post-transcriptional regulation to modulate mRNA stability by duplexing with the 3′ UTRs of mRNAs. Prominent among these cases are a group of lncRNA stemmed half-STAU1-binding site RNAs (½-sbsRNAs) that transactivate Staufen1-mediated mRNA decay via forming imperfect base pairs with targeted mRNA [36,37].

### 2.4. Translational Regulation

LncRNAs can also bind to the translational machinery, thereby modulating gene translation. For instance, lincRNA-21 pairs with specifically CTUNNB1 and JUNB mRNAs recruit the translation repressor Rck, which inhibits translation through a mechanism that induces ribosome drop-off [38]. Moreover, the antisense ubiquitin carboxyl-terminal hydrolase L1 (Uchl1) is abounded in the nucleus, whereas shuttled to the cytoplasm when subjected to rapamycin, a universal translation inhibitor. The decline in lincRNA-p21 level in turn negatively regulates JunB and β-catenin translation, and a corresponding decrease in the levels of these proteins was observed. Antisense Uchl1 RNA is then essential for the interaction between the overlapping sense protein-coding mRNA and active polysomes for translation under certain stress conditions [39].

### 2.5. Post-Translational Modification

Post-translational modification, encompassing the covalent and generally enzymatic modification of proteins following their biosynthesis, commonly occurs in cell signaling, as when phosphorylation of a protein modulates its activation state, and ubiquitination of a protein contains particular degradation signals [40,41,42]. In this regard, lncRNAs can disturb the interaction between proteins and protein-modifying enzymes, such that hinders post-translational modifications, thus affecting critical signaling pathways. Investigation concerning conventional dendritic cells exclusively expressed transcripts lnc-DC showed that this lncRNA interacts with signal transducer and activator of transcription 3 (STAT3) in the cytoplasm. In this context, lnc-DC sequester STAT3 from an SH2 domain-containing protein tyrosine phosphatase 1, thus promoting STAT3 phosphorylation on tyrosine 705 and further allowing the activation of STAT3 signaling [43]. Furthermore, NKILA, an NF-κB-induced lncRNA, directly interact with NF-κb/IκB to form a stable NKILA: NF-κB: IκBα ternary complex in the cytoplasm and inhibits IκB phosphorylation by masking the IfARrKK phosphorylating sites, which in turn inactivates NF-κB [44].

The complex physiology of eukaryotic cells is regulated by several multilayered and interconnected mechanisms. The increasing body of research proposes that lncRNAs participate in nearly every biological process, especially by acting as regulators of gene expression. As the comprehension of lncRNA mechanisms progresses, it expands on the spectrum of gene expression regulation, as well as other biological progress.

## 3. LncRNAs in Renal Interstitial Cell, Podocyte, Mesangial Cell and Epithelium

**Figure 2:**
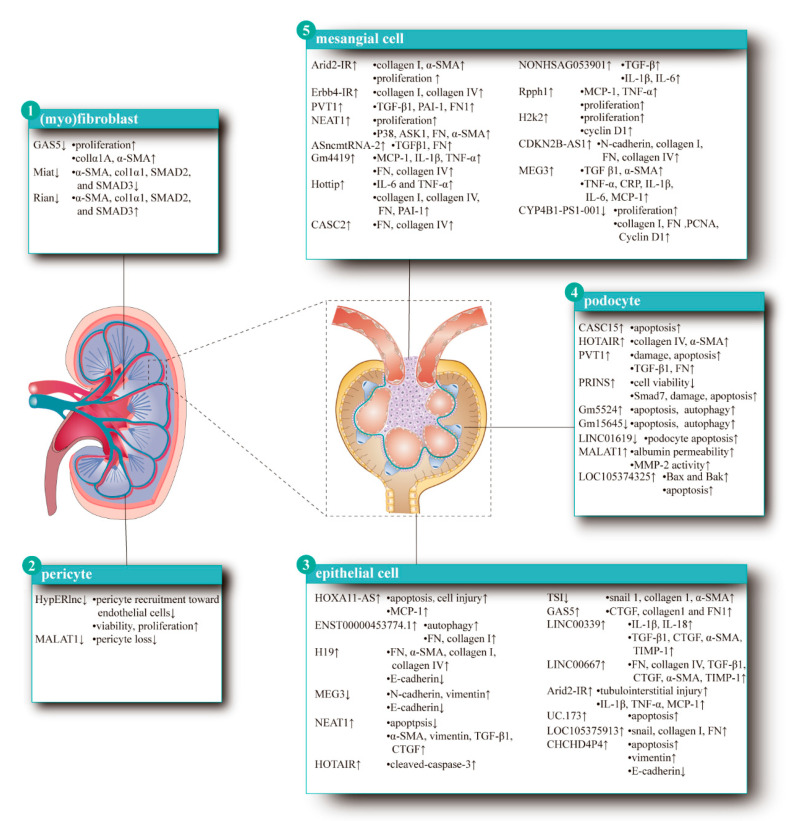
LncRNAs in cells of kidney. LncRNAs regulate cell viability, proliferation, migration, activation, autophagy, and apoptosis, as well as facilitates the production of ECM. (Myo) Fibroblast: Down-regulated GAS5 promotes proliferation and the expression of collagen α1A (collα1A) and α-SMA. Down-regulated Miat decreases the expression of α-smooth muscle actin (α-SMA), collα1, SMAD2, and SMAD3 while the silencing of Rian causes the opposite effect. Pericyte: The knockdown of HypERlnc increases cell viability and proliferation, but decreases cell recruitment. Pericyte loss appeared along with the silence of MALAT1. Podocyte: CmASC15 up-regulated and LINC01619 down-regulated increase apoptosis. Up-regulation of HOTAIR increases the expression of collagen IV and α-SMA. Overexpression of PVT1 promotes cell damage, apoptosis, and the expression of TGF-β1 and fibronectin (FN). PRINS up-regulated decreases cell viability, but increases Smad7, cell damage, and apoptosis. Apoptosis and autophagy are increased because of up-regulated Gm5524 and down-regulated Gm15645. MALAT1 up-regulated increases albumin permeability and matrix metalloproteinases-2 (MMP-2) activity. LOC105374325 up-regulated increases apoptosis, as well as the expression of Bax and Bak. Mesangial cell: Up-regulated Arid2-IR promotes collagen I, α-SMA, and proliferation. Erbb4-IR up-regulated increases collagen I and collagen IV. Overexpression of PVT1 increases the amount of TGF-β1, plasminogen activator inhibitor-1 (PAI-1), and FN1. Cell proliferation, P38, apoptosis signal-regulating kinase 1 (ASK1), FN, and α-SMA are promoted by NEAT1 up-regulating. Up-regulated ASncmtRNA-2 increases TGF-β1 and FN expression. Overexpressed Gm4419 increases monocyte chemoattractant protein-1 (MCP-1), IL-1β, TNF-a, FN, and collagen IV expression. Overexpression Hottip increases the amount of IL-6, TNF-a, collagen I, collagen IV, FN, and PAI-1. Up-regulated CAS2 increases the protein level of FN and collagen IV. Up-regulated NONHSAG053901 increases the amount of TGF-B, IL-1β, and IL-6. MCP-1, TNF-a, and cell proliferation are increased because of overexpressed Rpph1. Cell proliferation and cyclin D1 are increased by overexpressed H2k2. CDKN2B-AS1 up-regulated increases the expression of N-cadherin, collagen I, FN, and collagen IV. Overexpressed MEG3 increases TGF-β1, a-SMA, TNF-β, CRP, IL-1β, IL-6, and MCP-1. The down-regulation of CYP4B1-PS1-001 increases cell proliferation and collagen I, FN, PCNA, and cyclin D1 expression. Epithelial cell: HOXA11-AS up-regulated increases apoptosis, cellular injury, and the expression of MCP-1. Increased ENST00000453774.1 promotes cell autophagy along with the expression of FN and collagen I. H19 overexpressed increases FN, α-SMA, collagen I, and collagen IV expression, but decreases E-cadherin. Up-regulated MEG3 increases N-cadherin and vimentin expression, but decreases E-cadherin. Increased NEAT1 restrains apoptosis, but promotes the expression of α-SMA, vimentin, TGF-β1, and connective tissue growth factor (CTGF). HOTAIR overexpression promotes cleaved-caspase-3 expression. Down-regulated TSI increases the expression of snail 1, collagen I, and α-SMA. GAS5 up-regulation increases CTGF, collagen I, and FN1 expression. LINC00339 overexpression increases IL-1β, IL-18, TGF-β1, CTGF, α-SMA, and TIMP-1 expression. LINC00667 up-regulated enhances the level of FN, collagen IV, TGF-β1, CTGF, α-SMA, and TIMP-1. Tubulointerstitial injury, the expression of TGF-β1, FN, collagen I, α-SMA, and phosphorylation of Smad3 are promoted by Arid2-IR up-regulating. Up-regulated UC.173 promotes apoptosis. Increased LOC105375913 enhances the expression of snail, collagen I, and FN. Up-regulated CHCHD4P4 increases apoptosis and vimentin while decreases E-cadherin expression.

Glomerular innate cells include endothelial cells, mesangial cells, and epithelial cells. They all can produce amounts of ECM, which includes type IV and type VI collagen, laminin, and fibronectin. Under pathological condition, matrix synthesis increases and deposits in mesangial area, which squeezes the capillary lumen and leads to the apoptosis of glomerular cells continuously. ECM will then fill in the gap that left by the apoptotic and exfoliated cells. Followed by endothelial injury, the basement membrane of capillaries is exposed, podocytes are damaged, and further selective filtration of the glomerulus is deprived [45]. The cytokines, chemokines, and growth factors, produced by the injured glomerular cells and inflammatory cells, promote interstitial inflammation and fibrosis [46]. LncRNAs can regulate cells in the kidney by the above-mentioned five mechanisms, which may, in fact, impact cells’ viability, proliferation, migration, activation, autophagy, or apoptosis. At the same time, lncRNAs can also facilitate the production of ECM by over-expressed fibrotic protein, including collagens and fibronectin (FN). In the following section, different cell masses in the kidney that related to lncRNAs will be represented to explain the effect of lncRNAs as illustrated in Figure 2.

### 3.1. LncRNAs and Renal Interstitial Cells

The renal interstitium, which was the extravascular and intertubular space of the kidney and filled with interstitial fluid and scattered cells, is obligatory for the transit of all substances from kidney tubules to vessels [47,48,49,50,51,52]. Predominant cell type fibroblast and its active form, myofibroblast, are the primary source of ECM proteins [2,53,54,55,56,57]. Besides, pericytes, also called resident fibroblasts, are significant in sustaining normal endothelial barrier function [58,59,60,61]. In the following, an update on the interaction between interstitial cells and lncRNAs in kidneys will be given, with the main emphasis on fibroblasts, myofibroblasts, and pericytes.

*Fibroblasts and Myofibroblasts:* The lncRNA (growth arrest-specific 5) GAS5, which can be down-regulated by TGF-β, is a well-studied regulator of fibroblasts activation and proliferation [62]. GAS5 can restrain the fibrotic process by binding to Smad3, thus, enhancing the combination of phosphatase PPM1A to Smad3, which accelerated Smad3 dephosphorylation, and the dephosphorylation, eventually, block the activation of fibroblasts, and the synthesize of collagen 1A and α-SMA. Additionally, GAS5 overexpression can blunted fibroblasts proliferation via the suppression of TGF-β-induced JNK phosphorylation. This research offers valuable insights into the brake nature of GAS5 in renal fibrosis; however, whether JNK and TGF-β/Smad3 signaling are independent requires future studies. LncRNA myocardial infarction associated transcript (Miat) knockdown repress kidney fibrosis, attends the expression of profibrotic genes, such as SMAD2, SMAD3, col1α1, and α-SMA [63]. The silencing of lncRNA RNA imprinted and accumulated in nucleus (Rian) demonstrates opposite effects and enhances the expression of myofibroblasts markers. Another lncRNA Erbb4-IR can be transcriptional up-regulated by TGF-β1 and may participate in fibrosis process by binding Smad3 protein with its upstream sequence and then regulate ECM expression [64]. Knockdown of Smad3 by its inhibitor SIS3 can blunt the TGF-β1-induced Erbb4-IR, which verified the above-mentioned mechanism [65]. The specific function of Erbb4-IR in fibrosis, however, needs more investigations. Erbb4-IR can also be induced by advanced glycation end products (AGE) also in fibroblasts. Its effects, however, needs more exploration [66].

*Pericytes:* Hypoxia, which further regulates profibrotic signaling pathways, can induce the expression of lncRNA hypoxia-induced endoplasmic reticulum stress regulating long non-coding RNAs (HypERlnc) in human pericytes [67]. Silencing of HypERlnc with LNA GapmeRs in pericytes reduces pericyte recruitment toward endothelial cells (pericytes were defined as recruited when attached to endothelial cells). Furthermore, knockdown of HypERlnc can decrease pericyte viability and cell proliferation. Studies also focused on the function of lncRNA MALAT1 in pericytes [68]. Diabetes rats induced by streptozocin (STZ) presented severe pericytes loss, while knockdown of MALAT1 by GapmeRs can reverse the diabetes-induced pericytes loss. However, the exact mechanism needs further research.

### 3.2. LncRNAs and Podocyte 

An increasing body of studies proposed that the apoptosis and/or autophagy of podocytes resulting from the aberrant expression of lncRNAs contributes to the damage of kidney during disease pathology and further causes renal fibrosis [69]. Chronic kidney diseases (CKD), such as diabetic nephropathy (DN), chronic glomerulonephritis, crystal related nephropathy, IgA nephropathy, etc., can cause a sharp drop in renal structure and function, and lead renal fibrosis at last. Under the condition of CKD, while the expression of lncRNAs is induced, matrix synthesis increases and deposits in the mesangial area, which squeezes the capillary lumen, then leads to the apoptosis of glomerular cells continuously. Because of the endothelial injury, the basement membrane of capillaries is exposed, and the podocytes are damaged. Therefore, the renal tissues are lack of glomerular selective filtration, accompanied by glomerular cells apoptosis, inflammatory activation of glomerular residual cells, and leukocytes infiltration. In the end, the renal tissues present fibrosis [45]. Up-regulation of lncRNA CASC15 was observed in plasma derived from diabetic patients complicated with chronic renal failure, thus indicating the potential diagnosis value of CASC15 for chronic renal failure. Later experiments confirmed a corresponding elevated expression of CASC15 in the human podocyte cell line CIHP-1 under high glucose (HG) conditions. The in-depth analysis demonstrated that CASC15 can sponge miR-43c from position 492 to 509, thus, attenuating the inhibition role of miR-43c on podocytes apoptosis [70]. Similarly, elevated level of lncRNA HOTAIR was observed in human podocytes subjected to HG stimuli, whereas neither significant change in albuminuria, glomerular structure, podocyte number and podocyte ultrastructure nor accelerated glomerular injury in diabetic condition was detected in podocyte-specific knockdown of HOTAIR mice in vivo. To further inspect into the exact mechanism of HOTAIR in kidney disease, Majumder and colleagues performed silico analysis and CHIP and demonstrated that the expression of HOTAIR was modulated by p65, a submit of NF-κB, in mice podocytes exposed to HG. Bioinformatic analysis of data, obtained from publicly accessible datasets, exhibits the correlated expression between HOTAIR and HOXC11 gene, a gene located near sequence of HOTAIR and dysregulated in CKD, thus indicating that HOTAIR in podocytes may participate in the development of CKD and even renal fibrosis through the regulation of HOXC11 in cis [71]. Besides, overexpression of lncRNA plasmacytoma variant translocation 1 (PVT1) was observed in mouse podocytes with DN, which further promotes podocytes injury and apoptosis. Additionally, podocytes injury and apoptosis modulated by PVT1 up-regulation promote the accumulation of TGF-β1 and FN1, regarded as critical cytokine and ECM component, respectively, in the pathogenesis of fibrosis [72,73]. Similarly, the expression level of lncRNA PRINS was increased in patients with DN, and further in vitro experiments revealed that overexpression of PRINS reduced cell viability and enhances Smad7 expression in mouse podocytes in high glucose medium, which participates in the apoptosis of podocytes and ultimately increases the damage of the kidney to accelerate fibrosis [74]. 

Apart from the aforementioned lncRNAs up-regulated during renal fibrosis, the expression of lncRNA LINC01619 was decreased in biopsy tissues obtained from a patient with DN, thus indicating the negative correlation between LINC01619 and the pathology of DN. In vitro experiments suggest that LINC01619 serve as miRNA sponges for miR-27a to worsen podocytes injury accompanied by ER stress. Both biological processes indicate a declined in renal function, which is a common consequence of renal fibrosis [75]. Intriguingly, dynamic expression of MATAL1, with a rise to decline, was observed in podocytes exposed to HG. There was an increase of MALAT1 expression in podocytes exposed to HG, peaking at 4 hours, with a subsequent reduction to the basic level comparable to podocytes in the control group. Further experiments demonstrated that the subsequent decline was correlated with the aberrant accumulation of β-catenin in nucleus. This, therefore, disturbed the feedback loop between MALAT1 and β-catenin in normal conditions, and culminated in accelerated podocytes injury and renal fibrosis [76]. Dysregulation of lncRNA GM15645 and GM5524 was found to participates in the HG-simulated podocyte apoptosis and autophagy, as shown by the up-regulation of Bcl2, cleaved caspase 3, Bax and LC3I and a corresponding down-regulation of LC3II, Atg5, Atg7 and Blc2 [77]. Similar results regarding the also reported on the lncRNA LOC105374325, which participates in renal fibrosis by modulating podocytes apoptosis [78].

### 3.3. LncRNAs and Mesangial Cell

Insights into the lncRNAs in the regulation of mesangial cell proliferation and ECM protein secretion have started to emerge over the past decades and sheds light on the progress of the fibrotic renal disease [79]. For instance, enriched expression of lncRNA AT-rich was observed in the mouse UUO model. Overexpression of lncRNA AT-rich in mouse mesangial cells contribute to the proliferation of mesangial cells and facilitate ECM components, such as collagen Ⅰ and α-SMA (α-smooth muscle actin) secretion, which collectively promote renal fibrosis [80]. LncRNA Erbb4-IR is significantly up-regulated in a Smad3-dependent manner in the advanced glycation end products (AGE)-treated mesangial cells, which represent a well-established DN model. Through dual-luciferase reporter assay loss-of-function experiment, researchers definitely demonstrated that in mouse mesangial cells, Erbb4-IR contact with the Erbb4-IR and the 3′ UTR of miR-29b genomic sequence to repress miR-29b expression at the transcriptional level. Silencing of renal Erbb4-IR exhibited a protective effect on the kidney from progressive renal injury in DN mouse models and prohibited mouse mesangial cells from AGE-induced repression of miR-29b and fibrotic response in vitro [66]. Intriguingly, recent research represented the repressive effects of lncRNA on mesangial cell activation. For instance, the up-regulation of lncRNA CYP4B1-PS1-001 inhibited mouse mesangial cell proliferation and fibrosis as shown by the aberrant expression of Collagen I, Cyclin D1 and FN [81]. However, whether other lncRNAs exist that exhibit a negative effect on fibrogenesis through the regulation of mesangial cell proliferation, further explorations are required.

Moreover, the mechanism of lncRNAs competitively binding to miRNAs is involved in the proliferation and the ECM secretion of mesangial cells. Through RNA sequencing and bioinformatic analysis, Chen and colleagues decipher the interaction between lncRNA and miRNA during the pathology of DN and definitely revealed a DN-related ceRNA network made up of 153 lncRNAs, 428 mRNAs, and 2242 interactions. Investigators then focus on the topological key RNA pair in the DN-related ceRNA network—which is consistent with the in vitro results of mesangial cells in HG conditions, the lncRNA H2k2-miR-449a/b-Trim11 regulation axis was proposed. Further experiments validated that in mesangial cells exposed to HG, H2k2 competitively binding to miR-499a/b, then, it facilitated the expression of Trim11, a protein that activates Mek/erk signaling pathway to promote mesangial cell proliferation [82]. Overexpression of lncRNA PVT1 induced by hyperglycemic conditions promotes the expression of primary components of glomerular ECM, including plasminogen activator inhibitor 1, and TGF-β1, FN1 in mesangial cells, thus indicating the positive role of PVT1 in renal fibrosis. The in-depth experiment revealed that PVT1 by serving as a miR-1207-5p sponge, which then competed with the negative effect of miR-1207-5p on TGF-β1, FN1 expression [73]. By acting as a miRNA sponge for miR-23c, lncRNA NEAT1 facilitates mesangial cells proliferation together with fibrosis, as shown by the increased levels of fibrogenic proteins, such as ASK1, P38, α-SMA, and FN [83]. Down-regulation of lncRNA CASC2 was also observed in mesangial cells exposed to HG. LncRNA CASC2 demonstrates negative effects on miR-133b to promote mesangial cells proliferation and ECM component expression [84]. Similarly, in human mesangial cells treated with HG, the overexpression of lncRNA CKN2B-AS1 promotes the expression of fibrotic markers, such as FN, collagen I, and collagen IV, by acting as an endogenous sponge for miR-424-5p [45]. Additionally, lncRNA ASncmtRNA-2 is up-regulated in diabetic kidneys and HG-treated mesangial cells. Up-regulation of lncRNA ASncmtRNA-2 can increase the expression of ECM-related proteins TGF-β1 and FN in HG-induced human renal mesangial cells, which lead to renal fibrosis [85]. 

Before the excess deposition of ECM, inflammatory response represents to be the early reparative cascades during normal wound healing and renal fibrotic disease. Intriguingly, overexpression of lncRNA Gm4419 in mesangial cells induced by HG treatment not only facilitate the secretion of ECM components FN and collagen IV, but also facilitate inflammatory response, as shown by the up-regulation of pro-inflammatory cytokines, such as monocyte chemoattractant protein-1 (MCP-1), TNF-α, and IL-1β. Further experiments elucidated that Gm4419 directly interacts with the p50 submit of NF-κB to promote its shuttle from the cytoplasm to the nucleus, which then facilitates NLRP3 inflammasome formation and Gm4419 transcription. These data collectively indicated that the potential role of Gm4419 in the pathology of renal fibrotic disease through the regulation of both ECM protein expression and inflammatory response [86]. Up-regulation of lncRNA Hottip induced by HG treatment can promote both mesangial cells proliferation and the expression of inflammation factors which then consist of the inflammatory milieu during tissue fibrosis, such as IL-6 and TNF-α [87]. Similarly, up-regulation of lncRNA NONHSAG053901 in was observed in DN mouse models and overexpression of this lncRNA aggravated the severity of DN, thus indicating the involvement of NONHSAG053901 in the pathology of DN. In vitro experiments explained that NONHSAG053901 directly interacted with EGR-1 and then elevated TGF-β activity, as a consequence, enhance the levels of inflammation, fibrosis, and proliferation in mesangial cells [88]. Furthermore, over-expression of lncRNA Rpph1 related with DN-related factor galectin-3 (Gal-3) directly up-regulated inflammation factors like Mcp-1 and TNF-α and proliferation via Gal-3/Mek/Erk signaling pathway in MCs with low glucose, which may further lead to renal fibrosis [89]. Another investigation verified that the up-regulation of lncRNA MEG3 promoted fibrosis and inflammatory response and MEG3 was found to be the sponge of miR-181a. Additionally, over-expression of MEG3 enhanced the expression of fibrosis-related protein TGF-β1 and α-SMA and raised the concentrations of inflammatory cytokines, such as TNF-α, CRP, IL-1β, IL-6, and MCP-1 through Egr-1/TLR4 signaling [90].

### 3.4. LncRNAs and Epithelium

Innate immune characteristics show that tubular epithelial cells (TECs) are immune responders and can respond to a wide range of insults, thus producing and releasing inflammatory factors and ECM, driving interstitial inflammation and fibrosis with the involvement of lncRNAs [91]. LncRNA HOXA11-AS was up-regulated in the human proximal tubular epithelial cell (HK-2 cell) exposed to calcium oxalate monohydrate (COM). Over-expression of HOXA11-AS can restrain proliferation, aggravate apoptosis and cell damage via regulating the expression of monocyte chemotactic protein 1(MCP-1) through sponging miR-124-3p [92]. Besides, the expression of lncRNA ENST00000453774.1 (74.1) was significantly down-regulated in the TGF-β-induced fibrosis process. At the same time, the study showed that lncRNA 74.1 took part in autophagy mediation. Relative mRNA expressions of autophagy-related genes like Atg5, Atg7, and Becilin1 were all remarkably increased along with lncRNA 74.1 over-expression. Meanwhile, further researches showed that lncRNA 74.1 over-expression respited renal fibrosis and alleviated ECM deposition like FN and collagen I [93]. Another lncRNA can promote the expression of FN, α-SMA, collagen I and collagen IV, down-regulate the expression of E-cadherin expression, and aggravate renal fibrosis via sponging miR-17 [94]. In another study, an HK-2 cell fibrosis model was created by TGF-β1-treatment and revealed a down-regulation of lncRNA MEG3. Then researchers demonstrated that the over-expression of MEG3 significantly increased the expression of E-cadherin, and accordingly decreased N-cadherin and vimentin. Moreover, MEG3 up-regulation restrained cell viability and proliferation [95]. Furthermore, in HK-2 cells, studies showed that up-regulated lncRNA NEAT1 could lead to the over-expression of the α-SMA, vimentin, and the TGF-β1 and connective tissue growth factor (CTGF), and significantly promote apoptosis [96]. Meanwhile, lncRNA CHCHD4P4 was up-regulated in response to calcium oxalate in HK-2 cells with the increased vimentin protein levels and decreased E-cadherin protein levels, which may further affect cell proliferation and caused apoptosis [96]. Moreover, the up-regulation of lncRNA HOTAIR in HK-2 cells can negatively regulate the expression of miR-22 and increase the level of lipopolysaccharides (LPSs). Moreover, previous studies showed that LPSs promoted high mobility group box 1(HMGB1) and apoptosis protein cleaved-caspase-3 expression, which leads to the HK-2 cell apoptosis and fibrosis [97]. It has been provided that lncRNA TSI was up-regulated in TGF-β1-treated human TECs (HK-2 and HKC8 cells), and was negatively correlated with fibrosis. UUO mice showed that lnc-TSI can function as an endogenous inhibitor of the TGF-β1/Smad3 pathway and decrease the expression of snail 1, collagen Ⅰ, and α-SMA [8] LncRNA GAS5 was up-regulated in the DKD mouse model and TGF-β1-induced HK-2 cells. The results displayed that GAS5 accelerates fibrosis by sponging with miR-96-5p, which increases mRNA and protein levels of CTGF, collagen Ⅰ, and FN1 [98]. In HK-2 cells, lncRNA LINC00339 over-expression significantly increased protein expression of the secretion of pro-inflammatory IL-1β and IL-18. In addition, LINC00339 over-expression leads to a significant increase in lactate dehydrogenase (LDH) secretion and the expression of CTGF, TGF-β1, α-SMA, and TIMP-1, which can promote cell pyroptosis by sponging miR-22-3p and lead to fibrosis at last [99]. Simultaneously, over-expression lncRNA LINC00667 could also promote fibrosis in chronic renal failure (CRF) through competitively binding to miR-19b-3p and increase ECM protein expressions of FN, collagen IV, TGF-β1, CTGF, α-SMA, and TIMP-1 [100]. In the lead-treated renal tubular epithelial cells, studies showed that up-regulated UC.173 had no influence on cell viability, cell cycle, and apoptosis—but decreased the lead-induced inhibition of HK-2 cell viability. On the contrary, down-regulated lncRNA UC.173 might aggravate cell apoptosis and finally cause fibrosis [101]. Furthermore, the activation of C3a/p38/XBP-1s pathway up-regulates lncRNA LOC105375913 in HK-2 and then increases the expression of ECM-related proteins, such as snail, collagen I, and FN [102]. In the UUO kidney, a Smad3-associated lncRNA Arid2-IR was remarkably up-regulated. Meanwhile, the up-regulated lncRNA Arid2-IR might promote renal inflammation (an up-regulation of IL-1β, TNF-α, and MCP-1) and injury via NF-κB without influencing TGF-β/Smad3-mediated renal fibrosis in UUO kidney model, as confirmed by the silencing of Arid2-IR, which did not alter the effect of TGF-β1 [103].

## 4. LncRNAs in Mesenchymal Cell Phenotype Transition

**Figure 3:**
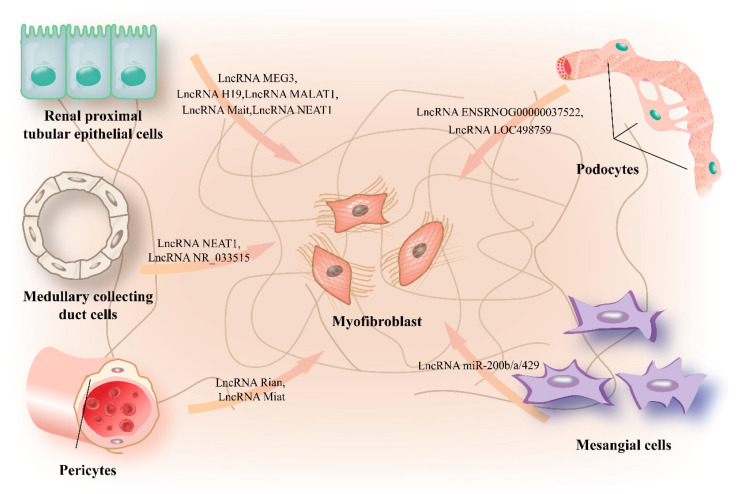
LncRNAs in mesenchymal cell phenotype transition. Diverse lncRNAs can participate in the pathologic renal fibrosis through regulating the acquisition of a fibrogenic myofibroblast phenotype of numerous cell types. The profibrotic or inflammatory microenvironment in injured renal tissue recruit and activate plenty of different cell types to acquire a matrix-producing myofibroblast phenotype: Renal proximal tubular epithelial cells, podocytes, medullary collecting duct cells, mesangial cells, and pericytes. Then, the activated myofibroblasts progressively secrete collagen and other extracellular matrix components in a positive-feedback loop that end up with the pathology of renal fibrosis.

Renal fibrosis generally occurs through the progressive accumulation and deposition of ECM secreted by myofibroblasts. The major source of these myofibroblasts has been a controversial issue over the past several years. Some investigator previously concluded that EMT or epithelial cell reprogramming—in other words, a biological process referring to the epithelial or endothelial cells phenotypic conversion into myofibroblasts, appear to be the predominant ancestors of myofibroblast during renal fibrosis. Although initial cell-fate tracing experiments support a substantial contribution of renal epithelial cells to myofibroblasts, lineage tracing in transgenic mice, however, demonstrates that resident stromal cells, including glomerular mesangial cells, interstitial fibroblasts and pericytes rather than epithelial-derived cells appear to be the predominant source of matrix-producing myofibroblasts [104,105,106,107,108]. Here, we provide up to date information on lncRNAs involved in mechanisms of the mesenchymal cell phenotype transition, as illustrated in Figure 3.

*Renal proximal tubular epithelium:* TGF-β is a multifunctional cytokine that has been accepted as the “main switch” in the induction of fibrosis in several tissues, including renal. The central role of TGF-β is to induce EMT, which may expound the continuous replenishment of fibroblasts in the process of tissue fibrosis [109,110]. Previous research illustrated that MEG3 expression was repressed in response to TGF-β1 simulation by regulating the methylation status of the CpGs positioned within MEG3 gene promoter region. The overexpression of lncRNA MEG3 prevents TGF-β1-induced EMT, as shown by the elevated expression of E-cadherin and correspondingly inhibited protein level of N-cadherin and vimentin in HK2 transferred with overexpression plasmids of MEG3, and therefore, rescues TGF-β1-induced renal fibrosis. LncRNA MEG3 might, thus, function as a candidate therapeutic target in renal fibrotic processes, though further investigation is still required [95]. The expression of H19 was elevated in HK2 cells upon TGF-β2 simulation in vitro, an action which then promotes the phenotypic transition of EMT as shown by limited levels of E-cadherin, increased levels vimentin and α-SMA and ECM proteins. Additionally, enriched expression of lncRNA H19 reduced miR-17 levels, whose overexpression can impede fibronectin expression, whereas H19 silencing represents opposite effects and alleviates TGF-β2-induced EMT^94^. Similarly, lncRNA MALAT1 serves as an endogenous miR-143 sponge to enhance protein Zinc Finger E-Box Binding Homeobox 2, a DNA-binding transcription factor involved in the expression of mesenchymal genes and E-cadherin, which further promotes EMT in renal fibrosis [111]. The lncRNA MALAT1 is a key regulator of the cellular processes modulated by TGF-β, such as EMT, ECM protein accumulation, cell growth and cell migration. In patients with obstructive nephropathy, lncRNA MATAL1 levels were positively related to hydronephrosis, together with ECM deposition and the expression of α-SMA, indicating that enriched expression of MALAT1 might lead to the renal fibrosis pathogenesis in patients with obstructive nephropathy. Elevated expression of MALAT1 was observed in TGF-β2 treated HK2 cells, which further induced EMT. Through dual-luciferase reporter assay and RNA immunoprecipitation analysis, investigators definitively demonstrated that MALAT1 functions as a miR-145 sponge to allow the expression of FAK, and the miR-145 mimics or silencing of FAK reverse the TGF-β2-induced EMT. Further experiments showed that the up-regulated expression of MALAT1 in TGF-β2-treated HK2 cells can be attributed, at list partly, to the m6A modification catalytic by methyltransferases METTL14 in vitro, an action that affect RNA stability and protein translation efficiency [112]. These data collectively demonstrated that m6A-induced lncRNA MALAT1 exacerbate renal fibrosis in obstructive nephropathy via the miR-145/FAK pathway [113]. To explore the function of lncRNAs in renal interstitial fibrosis, investigators examined transcriptional changes during renal interstitial fibrosis and identified a cluster of lncRNA Mait, which has a central role in renal interstitial fibrosis by facilitating EMT. Researchers found that Mait expression was significantly higher in both human and mice renal fibrotic tissues than in tissues of healthy individuals. Increased expression of Mait was observed in the renal proximal tubular epithelial cell line HK2 upon TGF-β1 simulation in a dose-dependent fashion in vitro. The pro-fibrotic effects of Mait are mediated through the interaction with miR-145 such that allow the expression of EIF5A2, a translation factor involved in cell growth, proliferation, and EMT [114].

Previous research has demonstrated that some signaling pathways, particularly the TGF-β/SMAD, Wnt/β-catenin, and ERK1/2 pathways, may induce EMT [114,115,116]. Additionally, besides acting as miRNA sponges, lncRNA can control EMT by impacting important signaling pathways at the post-translational level. In this regard, MALAT1 facilitates the β-catenin transfer from the cytoplasm to the nucleus, thus activate the Wnt/β-catenin pathway, which is implicated in the renal injury and the subsequent fibrotic response [117]. The overexpression of MALAT1 resulted from the HG condition induces EMT in HK2 cells. Further experiments demonstrated that compared with the HG group, MALAT1 deficiency impairs the HG-induced increase in cyclin D1, c-Myc, and β-catenin and that partly inhibited the β-catenin shuttle from the cytoplasm to the nucleus. Activation of the Wnt/β-catenin pathway promotes the EMT process with decreased expression of E-Cadherin a corresponding increase of α-SMA even though MALAT1 was knocked down. MALAT1 is, thus, considered to function at the post-translational level in the context of EMT via targeting regulating Wnt/β-catenin pathway activation [118]. NEAT1 presents another example of a lncRNA that regulates EMT via the post-translational modifications of the signaling pathway component. NEAT1 expression was repressed by klotho, an antiaging protein that intensely expressed in renal tubular tissue whose expression was decreased in the diabetes mellitus mice model during the progression of DKD. Increased NEAT1 expression was observed in HK2 cells in response to bovine serum albumin (BSA) culture that mimics DKD in vitro, which then induces EMT and fibrosis with enhanced cell migration accompanied by increased expression of vimentin and α-SMA. Through transfection with si-NEAT1 in the absence or presence of BSA, investigators definitively demonstrated that NEAT1 silencing not only directly prevents the ERK1/2 phosphorylation, but also inverts the ERK1/2 phosphorylation resulting from BSA stimulation in HK-2 cells. These data suggested that NEAT1 exerts its function in HK-2 cell fibrosis and EMT partly through the ERK1/2 pathway [119]. 

Some lncRNAs have potentially participated in the modulation of EMT transcription factors, including the Snail and ZEB families, which are regarded as the basic participants of EMT [120]. For example, the up-regulation of CHCHD4P4 induced by calcium oxalate monohydrate promoted mesenchymal-like characteristics in the HK-2 cells and improved the transcript levels of ZEB1, Vimentin, and Snail, whereas the silencing of CHCHD4P4 represented the opposite results. However, the exact molecular mechanism by which CHCHD4P4 regulates EMT-TFs expression remains enigmatic [121]. Studies have also linked other lncRNAs with EMT in renal fibrosis, but the underlying biological mechanisms involved in these lncRNAs regulation requires further investigation [122]. Additionally, an emerging consensus suggests that epithelial-derived cell hints at a central role for promoting fibrosis, but they only constitute a minor portion of myofibroblast pool [123,124]. At this point, a growing body of literature highlights the concept of epithelial cell reprogramming rather than EMT to describe these cellular fibrotic remodeling events [125,126,127,128]. Further studies are required to clearly define exact mechanisms by which lncRNAs anticipate epithelial cell reprogramming during fibrosis. 

*Podocyte:* Podocytes refer to the terminally differentiated visceral epithelial cells that wrap themselves around the glomerular capillaries to makeup the filtration slits. Emerging research demonstrates that excepting renal tubular epithelial cells, podocytes undergo EMT after injury, thereby causing the podocyte dysfunction—which ultimately results in defective glomerular filtration and renal fibrosis [129,130]. Li Ling and colleagues performed a microarray profiling analysis to identify lncRNAs associated with EMT during renal fibrosis. Using RNA isolated STZ-induced DN rats, they identified seven lncRNAs, including lncRNA ENSRNOG00000037522 and LOC498759, which were produced in a developmentally regulated manner throughout renal fibrosis. Silencing of ENSRNOG00000037522 reversed the HG-simulated EMT process with decreased expression of α-SMA and vimentin accompanied by a corresponding increase in podocyte-specific protein PODXL1 and nephrin, suggesting that ENSRNOG00000037522 participates in the modulation of podocytes in DN by facilitating EMT [131]. The latter study demonstrated that lncRNA LOC498759 is increased in HG-simulated podocytes. The overexpression of LOC498759 might be attributed to activating endogenous TGFβ/SGK1 signaling, a pathway considered to play a critical role in EMT and fibrosis. LncRNA LOC498759 deficiency impaired the HG-induced EMT as shown by the decreased expression of mesenchymal markers desmin and vimentin, as well as a correspondingly enhanced epithelial markers nephrin and P-cadherin protein levels. In contrast, overexpression of lncRNA LOC498759 abrogated the Jixuepaidu Tang-1-mediated renal protection in HG condition, and therefore, diminished the Jiexuepaidu Tang-1-mediated inhibition of podocytes EMT and alleviation of glomerular mesangial extracellular matrix accumulation [132].

*Medullary collecting duct epithelium:* A pre-mi-lncRNA encoding the miR-200b/a/429 cluster expressed in medullary collecting duct cells, and it is implicated to plays a role in EMT and further the polycystic kidney disease pathogenesis. Indeed, expression of the lncRNA transcript was specific to the kidney and RT-PCR analysis revealed that this pre-mi-lncRNA transcribed from the HNF-1β-dependent promoter to encode the miR-200b/a/429 cluster rather than its Ttll10 host gene. miR-200b/a/429 cluster suppressed the expression of Zeb2 and Pkd1, which has been implicated as a cystic disease gene and a transcriptional repressor for EMT progression, respectively. Collectively, these results identify a lncRNA transcript processed to produce the miR-200b/a/429 cluster, which may be involved in phenotypic abnormalities during polycystic kidney disease development. Further, these findings demonstrate that the miRNA that regulates important cell functions can be concealed within lncRNAs such that adds another layer of complexity to lncRNA in EMT [13].

*Mesangial cell:* LncRNAs that are known to promote mesangial cells EMT include NEAT1 and NR_033515. The expression of lncRNA NEAT1 was increased in the serum of DN patients and mice mesangial cells induced by a high concentration of glucose. NEAT1 promotes EMT by acting as a sponge for miR-27b-3p and culminating in the increased expression of ZEB1, which has been identified as a strong epithelial repressor and weaker mesenchymal promoter to induce pathologic of renal fibrosis. Notably, the direct association between NEAT1 and miR-27b-3p as well as miR-27b-3p and ZEB1 were confirmed by utilizing the dual-luciferase reporter assay and RNA immunoprecipitation experiments [133]. Besides targeting miR-27-3p and ZEB1, lncRNA NEAT1 also demonstrates negative effects on miR-23c, whereas the inhibition of miR-23c counteracted the suppressive effect of NEAT1 depletion on proliferation, fibrosis, and EMT of mice mesangial cells induced by high concentration glucose [83]. In the case of lncRNA NR_033515, Gao et al. demonstrated that NR_033515 is enriched in the serum of DN patients compared with healthy individuals. Moreover, the high expression of NR_033515 in DN patients down-regulates miR-743b-5p and attenuates its control on α-SMA, E-cadherin, Vimentin, leading to EMT in DN. NR_033515 is, thus, considered to function at the post-transcriptional level in the biological progress of EMT via targeting miR-743b-5p [134].

*Pericyte:* The genetically labeled pericytes and their myofibroblast derivatives in FoxD1-GC;tdTomato mouse models were then isolated from healthy kidneys and kidney injured by UUO or ischemia-reperfusion injury through fluorescence-activated cell sorter [135]. Quantitative PCR for myofibroblasts markers from isolated cell groups corroborated their acquisition of a myofibroblast phenotype, as demonstrated by elevated protein level of collagen-1α1 and α-SMA. Rian and Miat are two conserved lncRNAs identified to differentially expressed in the Tomato red-positive FoxD1-derivative cells of injured kidneys. Through loss-of-function approaches with antisense gapmers that silence Rian or Mait in the NIH3T3 mouse fibroblasts simulated by TGF-β, Roel Bijkerk and colleagues definitively demonstrated the regulatory role of Rain and Mait in myofibroblasts formation. Moreover, assessment of the miRNA profile in FoxD1-derivative interstitial cells uncovers dysregulation of miR-150 and 14q32 miRNA cluster. These clsuters were previously identified to interact with Miat and Rian, respectively, indicating a potential interaction between miRNAs and these lncRNAs in myofibroblasts formation. Collectively, these data suggest that Rian and Mait participate in myofibroblasts formation, probably through miRNA regulation [63]. 

## 5. LncRNAs in Vascular Damage and Rarefaction

**Figure 4:**
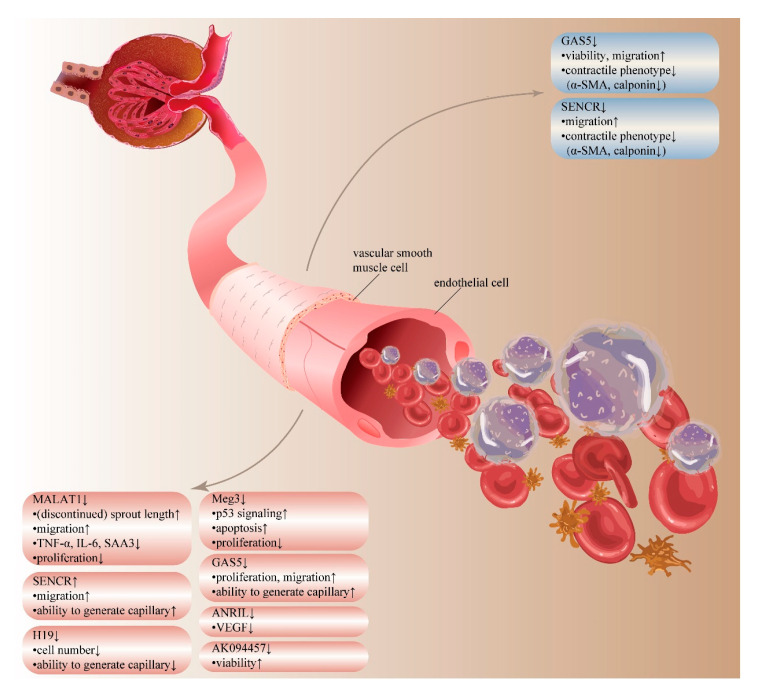
LncRNAs in vascular damage and rarefaction. LncRNAs regulate cells involved in the physiological and pathogenic processes of kidney vessels and capillaries. Endothelial cells: The silencing of MALAT1 results in more migration, angiogenic sprout length, discontinued sprout length, and repress the proliferation as well as the expression of TNF-α, IL-6, and serum amyloid antigen 3 (SAA3). The increase of SENCR promotes migration and the ability to generate capillary. The knockdown of H19 decreases cell number and suppress the ability to generate capillary. Decreased Meg3 promotes p53 signaling and apoptosis, and inhibits proliferation. Down-regulation of GAS5 increase the proliferation, cell migration, and capillary formation. The loss of ANRIL prevents VEGF up-regulation. AK094457 knockdown enhanced cell viability. Vascular smooth muscle cells (VSMCs): GAS5 knockdown increases cell viability, migration, and down-regulated the expression of α-SMA and calponin. The knockdown of SENCR inhibits the contractile phenotype of VSMCs and up-regulate migration.

Vessels carry metabolites and oxygen to tissue and export waste to maintain the well-being of kidney [136,137]. Apart from the classical barrier role, endothelial cells participate in many physiological or pathological processes like the regulation of vascular remodeling and the response to inflammation [138,139,140,141,142]. Kidney peritubular capillaries are especially susceptible to various damage [143,144,145,146]. Moreover, during the process of fibrosis, excessive ECM reduces peritubular blood flow, triggering tubular hypoxia and microvascular rarefaction, forming a self-reinforcing vicious cycle [147]. Although advances have been made to understand vascular damage and rarefaction, lncRNAs have added another layer of complexity to the regulation network, as illustrated in Figure 4. 

Several reports have shown that certain lncRNAs are essential in the microvascular hypoxic response. Studies have evaluated the function of lncRNA MALAT1 in endothelial cells. By using the siRNA-mediated or the GapmeR-mediated silencing of MALAT1, researchers demonstrated how MALAT1 inhibition could induce angiogenic sprouting and migration but repress proliferation of human umbilical vein endothelial cells (HUVECs). However, the MALAT1 silence group with more sprout length also has more discontinued sprout length [148]. Based on the model of unilateral renal ischemia-reperfusion (I/R) injury mice, MALAT1 was shown to be transcriptionally activated by hypoxia-inducible factor 1-α (HIF1-α). However, a disease-specific performance of MALAT1 knockout mice was not observed [149]. MALAT1 silencing and wild type group showed similar levels of capillary rarefaction. These studies may suggest that the effects of MALAT1 could be cell- or tissue-specific. Knockdown of another lncRNA H19 in HUVECs can induce a significant reduction of cell number and decreased the ability of HUVECs to generate capillary-like structures, while the overexpression of H19 by lentivirus infection showed no significant alteration of proliferation rate or capillary-like structure formation [150].

Vascular remodeling and vasculogenesis are active processes of structural changes [151]. LncRNAs are crucially involved in this procedure, which contribute to increased peripheral resistance. As an example, the long non-coding RNA GAS5 knockdown in spontaneously hypertensive rats increased the medial thickness and medial-tolumen ratio of the renal artery but did not affect luminal diameter [151]. GAS5 knockdown can increase the viability and accelerate the migration of vascular smooth muscle cells (VSMCs), down-regulated the expression of VSMCs markers, including α-smooth muscle actin and calponin as well as increase the proliferation ability, cell migration speed and tube formation of HUVECs by reducing the GAS5-β-catenin interaction. Interestingly, endothelial cells also can increase GAS5 levels in VSMCs via GAS5-contained exosomes. Another lncRNA SENCR was enriched in VSMCs, and the knockdown of which can inhibit the contractile phenotype of VSMCs and up-regulate genes promote migration [152]. Additionally, SENCR was also shown to regulate endothelial cell function and differentiation [153]. The pro-angiogenic and the promigratory ability of SENCR on endothelial cells suggests a potential role in renal vascular damage and fibrosis. Researchers used angiotensin II-stimulated HUVECs to mimic vascular endothelial injury [154]. LncRNA AK094457 knockdown enhanced the cell viability of HUVECs by up-regulating the expression of eNOS and p-eNOS. Analogously, lncRNA sONE can regulate the expression of eNOS, though the mechanism is unclear [155].

DN is a significant cause of CKD, which is characterized by vascular affections and progressive renal tubulointerstitial fibrosis [156]. MALAT1 overexpression can significantly promote the high glucose (HG)-induced injury in human renal glomerular endothelial cells (HRGECs), including increased levels of ROS and inflammatory factors (TNF-α and IL-6) through the combination of MALAT1 to G9a—thus, elevating H3K9me1 (lysine 9 of histone H3 monomethylation) and it’s binding to the klotho promoter [157]. The loss of another lncRNA ANRIL obviously prevented diabetes-induced vascular endothelial growth factor (VEGF) up-regulation by decreasing the epigenetically regulation of ANRIL on p300 and enhancer of zeste 2 (EZH2) [158]. Data from another study showed an increase in serum amyloid antigen 3 (SAA3), an inflammatory ligand that can be targeted by MALAT1, after HG treatment. Also, an up-regulation in inflammatory mediators, TNF-α, and interleukin 6 (IL-6) was observed, which can be prevented by the knockdown of MALAT1 [159].

Vascular damage and kidney fibrosis can also be triggered by drugs and toxins. Overexpression of MANTIS significantly reversed the I-bound P-cresol-induced inhibition on HUVECs viability, migration, and invasion, while the knockdown of MANTIS had opposite effects. This may be related to the interaction of MANTIS with Sox18, a key role in promoting the vascularization of HUVECs, as researchers observed a markedly up-regulation of Sox18 in MANTIS overexpression group [159].

LncRNA can also participate in this process by regulating DNA damage response. As an example, researchers explored the role of Meg3, a lncRNA regulated by p53-mediated transcription [160]. Meg3 silencing reduces the binding of Meg3 to polypyrimidine tract binding protein 3 (PTBP3), accounting for increased breaks of DNA double-strand in HUVECs. The silencing can also activate p53 signaling, increase the of p53 target gene expressions, accelerates HUVECs apoptosis, and inhibits HUVECs proliferation.

## 6. LncRNA-Based Diagnostics and Therapeutics Targeting Renal Fibrosis

*Risk prediction:* LncRNA expression levels may suggest disease risk. Researchers newly termed a lncRNA as TGF-β/Smad3-interacting long non-coding RNA (lnc-TSI) [8]. In a cohort of 58 IgA nephropathy (IgAN) patients confirmed by biopsy, researchers found a negative correlation between renal fibrosis index with the lnc-TSI renal expression (r = −0.56, *p* < 0.001). Also, a longitudinal study (an average of 48 months of follow-up) involved 32 IgAN patients whose renal lnc-TSI expression were in a low level presented serious kidney dysfunction and notably increases in the renal fibrosis index, as compared with patients whose baseline lnc-TSI levels were above or equal to the median. Thus, considering the correlation between lnc-TSI and renal fibrosis index, lnc-TSI could be a promising risk predictor of kidney fibrosis. 

*Diagnostic markers:* Recently, researchers have detected the presence of various lncRNAs inside the exosomes, which can also present in various body fluids, such as blood or urine [161]. Researchers have demonstrated the detectable of lncRNAs associated with renal fibrosis in urine [162]. Detection of these lncRNA uncovers the potential for using as markers and may promote the development of noninvasive diagnosis of kidney fibrosis. A study derived 25 urinary exosomes ncRNA profiles (15 from patients with CKD, 10 from healthy controls) and identified 30 statistically significant ncRNAs, including lncRNAs, which may serve as biomarkers for early diagnosis of CKD and renal fibrosis [163].

*Therapeutic potential targeting*: Certain lncRNAs may provide a novel strategy for fibrosis therapy, as summarize in Table 1. Therapeutic approaches like antisense oligonucleotides (ASOs) targeting lncRNAs have shown promising results in combating fibrosis [164,165]. Besides, lncRNAs silencing by using duplex RNAs can also be efficient [166]. Researchers used locked nucleic acid (LNA)-modified GapmeRs to knockdown a lncRNA, lnc-MGC. This further prevented podocyte death and glomerular fibrosis in diabetic mice. Similar results were detected in human mesangial cells, highlighting the potential clinical significance [12]. Knockdown of another lncRNA Erbb4-IR by shRNA or siRNA largely relieved renal injury, decreased serum creatinine and microalbuminuria excretion, and significantly inhibited the fibrotic responses, including expressions of TGF-β1, collagen I, and collagen IV as well as the phosphorylation of Smad3 [66]. Sometimes, instead of blocking lnRNAs, overexpression of lncRNAs can also yield beneficial effects. A study used plasmid transfection to up-regulate lncRNA NR_038323, which directly interacts with miR-324-3p to induce dual-specificity protein phosphatase-1 expression, thus to inactive p38MAPK and ERK1/2 pathways, playing an anti-fibrotic role [156]. Overexpression of lncRNA lnc-TSI and 1700020I14Rik also presented the inhibition of renal fibrosis [8,62]. These results provide initial support on targeting lncRNAs for treating renal fibrosis.

## 7. Conclusions and Future Perspectives

Renal fibrosis is characterized by excessive accumulation and deposition of ECM in the renal parenchyma, which is the common final result of virtually all chronic and progressive kidney diseases. Insights into the roles of lncRNAs in kidney disease have started to emerge over the last two decades. Distinct to other types of ncRNAs, lncRNAs display an amazingly wide range of sizes, shapes, and functions and usually poorly conserved across species. The aforementioned natures have endowed them with previously unanticipated functional capabilities; yet, these have also presented obstacles to their analysis and translation of findings in animal models to humans. Recent research adopting robust approaches has significantly expanded our knowledge of lncRNA functions. This review hammered to clarify the biological relevance of lncRNAs in renal fibrosis and its related pathogenesis as novel relevant regulators and potential therapeutic targets. Similar to proteins, lncRNAs participate in gene expression by versatile mechanisms of action, which then enable lncRNAs with various functions to regulate biological processes of kidney cells, mesenchymal cell phenotype transition and vascular damage and rarefaction during renal fibrosis. Therefore, lncRNAs unleash their potential as powerful tools for diagnostic and therapeutic use in the fields of risk prediction, diagnostic markers and therapeutic target during renal fibrosis. However, several current studies of lncRNAs in renal fibrosis merely illustrate the participation of lncRNAs in the pathogenesis of renal fibrosis instead of penetrating their specific role or potential specific mechanisms. Moreover, the application of lncRNAs as drug targets and therapeutic implement is still in its start-up step. Future studies will probably shed light on the mechanism and opportunity of renal fibrosis in the following aspects:

(I) Insight into the biological mechanism of lncRNAs would be augmented by encouraging by the development of sequencing resolution and the burst of novel technologies, which then advances our understanding of normal physiology and disease, including renal fibrosis.

(II) An in-depth understanding of the association of lncRNAs with renal fibrosis processes could also be augmented by heartening replication researches and collaborations, such as submission of discoveries, including sequence information and chromosomal location, to publicly accessible datasets. Important lncRNA databases are NONCODE, LNCiPedia, lncAtlas, lnc2Meth, InCeDB, NeuraNetL2GO, lncRNA Ontology, MFLDA, and lncRNADisease.

(III) Delineating lncRNA expression patterns of tissue-specific or cell-type-specific lncRNAs, which present to be diagnosis marker or therapeutic target, should be warranted. Such advances will foreshadow the occurrence of targeted therapy for renal fibrosis. For example, a study detected an acute kidney injury (AKI)-related lncRNA TrAnscript Predicting Survival in AKI (TapSAKI) [167]. Researchers then confirmed this lncRNA as an independent predictor of 28-day survival in AKI patients. Interestingly, TapSAKI was found to be specifically enriched in tubular epithelial cells rather than renal endothelial cells, which may suggest the origin of TapSAKI and guide further exploration. Another study found some CKD-related lncRNAs in urinary exosomes were secreted by renal proximal tubular epithelial cells, which act as primary sensors under stress situations [163,168]. This provides a functional perspective on lncRNA markers and targets.

(IV) An emerging consensus suggests that non-coding SNPs located in regulatory or functional elements have the potential to modulate gene expression in trans by means of long-range genome interactions. Additionally, SNPs within lncRNA-encoding genes may alter the expression or function of lncRNAs. Further inspection is desirable to uncover the mechanism by which these lncRNA-associated locus lead to renal fibrosis pathogenesis, which then expands our knowledge of this disease. Step forwards in genome-editing technologies, such as CRISPR/Cas9 and gapmer silencing, offers promise to inspect the participant of a certain lncRNA-associated SNP during renal fibrosis.

## Figures and Tables

**Table 1:** Representative therapy strategies for targeting lncRNAs.

Strategy	Chemistry	Mechanism of Action	Refs
Down-regulation	ASO gapmers	An ASO consists of a central DNA region flanked by modified nucleotides; the DNA-RNA hybrid recruits ribonuclease H and promotes degradation of target mRNA	[12]
	siRNA	siRNA gets incorporated into other proteins to form the RISC; siRNA is unwound and find a complementary mRNA and RISC induces mRNA cleavage	[66]
	shRNA	Ribonuclease protein Dicer binds and cleaves shRNA into siRNA; siRNA gets incorporated into other proteins to form the RISC; siRNA is unwound and find a complementary mRNA and RISC induces mRNA cleavage	[66]
Up-regulation	Adeno-associated virus	Insert the full length of lncRNAs into vectors; target cells uptake these vectors and up-regulates lncRNA expression	[8,62,156]

ASO, antisense oligonucleotide; siRNA, small interfering RNA; shRNA, short hairpin RNA; RISC, RNA-induced silencing complex.
